# In Vitro Activity of Rifampicin Combined with Daptomycin or Tigecycline on *Staphylococcus haemolyticus* Biofilms

**DOI:** 10.1007/s00284-015-0821-y

**Published:** 2015-04-18

**Authors:** Ewa Szczuka, Katarzyna Grabska, Adam Kaznowski

**Affiliations:** Department of Microbiology, Faculty of Biology, Institute of Experimental Biology, Adam Mickiewicz University, ul. Umultowska 89, 61-614 Poznan, Poland

## Abstract

*Staphylococcus haemolyticus* is of increasing concern as a cause of several biofilm-associated infections, and today, it represents the second most common organism among clinical isolates of coagulase-negative *staphylococci*. However, little is known regarding the treatment of infections caused by these bacteria. In this study, we characterize the biofilm formed by *S.haemolyticus* strains isolated from bloodstream infections and assess in vitro the activity of rifampicin combined with daptomycin or tigecycline against bacteria growing in a biofilm. The results of our studies indicated that the majority (78 %) of methicillin-resistant *Staphylococcushaemolyticus* strains have the ability to form a biofilm in vitro. None of these strains carried *icaADBC* genes indicating that they form biofilm via *ica*-independent mechanisms. The molecular characterization of the biofilm showed that proteins are the predominant matrix component and play a major role in biofilm structure. Extracellular DNA and polysaccharides, other than polysaccharide intercellular adhesin, are also present in the biofilm matrix, but they play a minor role. The images obtained by confocal laser scanning microscopy showed that most *S. haemolyticus* strains formed a dense biofilm with a low number of dead cells. In vitro study demonstrated excellent activity of tigecycline in combination with rifampicin against cell growth in the proteinous biofilm. The BIC (biofilm inhibitory concentration) value for tigecycline/rifampicin ranged from 0.062 to 1 µg/ml, whereas for daptomycin/rifampicin from 0.125 to 2 µg/ml. These results indicated that the tigecycline/rifampicin combination was more effective against *ica*-independent biofilm, formed by *S. haemolyticus* strains, than the daptomycin/rifampicin combination.

## Introduction

*Staphylococcus haemolyticus* belongs to a group of coagulase-negative *staphylococci* (CoNS) and is a skin commensal, as well as a significant pathogen, with the potential to cause persistent infections, including native valve endocarditis, prosthetic join infections, meningitis, skin and soft tissue infections as well as catheter-related bloodstream infections [5, 9, 16, 20]. Currently, *S. haemolyticus* is ranked as the second highest species of coagulase-negative *staphylococci* in importance among clinical isolates. This bacterium has the ability to induce apoptosis within host macrophages and also exhibits a cytotoxic effect towards murine macrophages cell lines J774 [10]. However, the virulence of CoNS is mainly attributed to the colonization of an inert, or living surface and biofilm formation. The biofilm represents an adherent, structured community of cells that is embedded in a self-produced, polymeric matrix [22]. The polysaccharide intercellular adhesin (PIA) is a linear homoglycan composed of at least 130 residues of β-1,6-linked *N*-acetylglucosamine as the major component of the matrix, and plays a crucial role in biofilm formation by *S. aureus* and *S. epidermidis*. PIA is the product of *icaADBC* gene expression. Other components, such as cell-wall associated proteins, extracellular DNA, teichoic acid and polysaccharide other than PIA, are capable of mediating intercellular accumulation and biofilm formation by *staphylococci* [22]. The biofilm growth mode of the bacteria protects them from the host’s immune system and antibiotics. Thus, biofilm-associated infections become chronic and their eradication by antibiotic therapy is quite rare [13, 21]. In addition, *S. haemolyticus* is frequently resistant to multiple antibiotics, including ß-lactams, and in such cases, only a few therapeutic alternatives are available. Glycopeptides, vancomycin and teicoplanin have been recommended as a first-line therapy for the treatment of methicillin-resistant *staphylococci* infections. Moreover, many reports described *S. haemolyticus* strains with a reduced susceptibility to teicoplanin [1, 26]. The disadvantage of vancomycin is its lack of activity against organisms growing in biofilm and poor tissue and intracellular penetration. Rifampicin has been shown to be one of the most potent in the eradication of *staphylococci* biofilm. However, rifampicin should not be used as a single agent to treat *staphylococci* infection, due to the rapid development of resistance [4]. Therefore, extensive studies regarding the use of the rifampicin, in combination with other antibiotics, should be considered in the treatment biofilm-associated infections. A recent study indicated that tigecycline, a glycylcycline antibiotic, exhibits excellent activity in vitro and in vivo against *S. aureus* strains [3]. Also, daptomycin, a novel cyclic lipopeptide antibiotic, with a unique mechanism of action against Gram-positive bacteria, and the low occurrence of spontaneous resistance against this antibacterial factor, has been successfully used for the treatment of bacteraemia caused by *S. aureus*. Furthermore, this antibiotic also demonstrated the ability to successfully penetrate into *S. epidermidis* biofilm [12, 23].

The purpose of this study was to characterize the biofilm formed by *S.haemolyticus* strains and assess in vitro the activity of rifampicin, in combination with daptomycin, and with tigecycline, against bacteria growing in a biofilm.

## Materials and Methods

### Bacterial Strains

Sixty-five *S. haemolyticus* strains were isolated from blood taken from in-patients at a hospital in Poznań, Poland. Only one isolate from one patient with bloodstream infections was included. Identification was performed using a Vitek 2 system (bioMérieux, France). All the isolates included in our studies were resistant to oxacillin. The resistance to methicillin was confirmed by the presence of the *mecA* gene, using the PCR method [7].

### Detection of the *ica* Operon

Chromosomal DNA was extracted using the Genomic DNA prep Plus kit (A&A Biotechnology, Gdynia, Poland). The PCR technique was applied to detect the presence of *icaADBC* genes in *S. haemolyticus* strains as described by Chokr et al. [2]. Amplification products were electrophoresed in 1.5 % agarose gel, stained with ethidium bromide and documented with a V.99 Bio-Print system (Vilber Lourmat, Torcy, France).

### Determination of Biofilm Production by Microtiter Plate Assay

To test the isolates for biofilm formation, a quantitative adherence assay was used [6]. Briefly, the overnight culture grown in a tryptic soy broth (TSB, Difco, Beckton Dickinson, France) supplemented with 0.25 % glucose was diluted 1:100 in TSB_glucose0,25 %_ and 100 µl was transferred into 96-well polystyrene microtiter plates. The plates were incubated overnight at 37 °C, then the medium was removed from each of the wells and the plates were gently washed three times, with phosphate-buffered saline (PBS) to remove non-adherent cells. Then, the biofilms in the wells were stained with a 0.4 % crystal violet solution. After washing, the crystal violet from the biofilm was dissolved using an ethanol–acetone mixture (70:30) and absorbance was determined. The strains were considered as biofilm positive if they had an OD_490_ > 0.250. Each isolate was tested in triplicate.

### Biofilm Detachment Assay

The biofilm in 96-well microtiter plates was rinsed three times with PBS and treated with 40 mM NaIO_4_ in H_2_O, or 0.1 mg/ml of proteinase K in 20mM Tris-HCl (pH 7.5) with 100 mM NaCl, or 0.5 mg/ml DNase (Sigma) in 5 mM MgCl_2_. After 24-h at 37 °C, the biofilms were stained with crystal violet as described above. The percentage of detachment was calculated on the basis of the average difference between the wells subjected to NaIO_4_, proteinase K, DNase and the control wells untreated with these factors. The detachment results were divided into three categories: no detachment (<10 %), intermediate detachment (10–50 %) and strong detachment (>50 %) [6].

### Confocal Laser Scanning Microscopy (CLSM)

Overnight cultures of various strains were inoculated into a coverglass cell-culture chamber (Nunc) [18]. After 24-h incubation at 37 °C, the chamber coverglasses were washed gently three times with PBS to remove planktonic cells. Bacteria in biofilms were stained using SYTO and PI (Live/Dead BacLight Bacterial Viability kits, Invitrogen) for 15 min, and observed under microscope (Carl Zeiss LSM 510- Axiovert 200M) with detector and filter sets for monitoring SYTO9, and PI Carl Zeiss confocal software was used for the analysis of the biofilm, which permitted the collection of image stacks and the three-dimensional (3D) visualization of the biofilms.

### Biofilm Susceptibility Assay

Bacterial biofilms were formed on a modified polystyrene microtiter lid (Nunc, TSP system), and the effect of antibiotics was determined according to the procedure described by Moskowitz et al. [15]. Briefly, 22-h-old bacterial cultures of each strain, in TSB with a 1 % glucose medium, were adjusted to a turbidity of 0.5 McFarland, and 100 µl of these cultures was transferred to wells of a 96-well microtiter plate covered with a 96-peg lid and incubated for a further 20-h, to allow biofilm development. The peg lids were rinsed three times in sterile water and placed into a new microtiter plate containing two-fold dilutions of antibiotics (e.g. 1024, 512, 256, 128, 64, 32, 16, 8, 4, 2, 1, 0.5, 0.25, 0.125, 0.062, and 0.030 μg/mL) and incubated for 18–20 h at 37 °C. The combination antibiotic solution contains the two agents in equal concentration. After this time, the biofilm cells were transferred by sonication from the pegs into recovery media, and the optical density at 650 nm was measured on a plate reader. The plate was incubated for six further hours, and the next bacterial growth was measured by optical density (OD_650_). Biofilm growth was defined as a mean OD_650_ difference (OD_650_ at 6 h minus OD_650_ at 0 h) ≥0.05 for the biofilm control. The BIC values were defined as the lowest concentration without growth.

## Results

Fifty-one (78 %) methicillin-resistant *S.haemolyticus* strains were able to adhere to polystyrene and to form a biofilm on this surface. None of these strains gave an amplicon in the PCR assays with primers designed for four different regions of *icaADBC* genes. We estimated the composition of the biofilm matrix, by treating the biofilm with proteinase K, NaIO_4_ or DNAse. Firstly, biofilms of *S.haemolyticus* were very effectively dispersed, when proteinase K was added to a 24-h biofilm. Thirty-six strains showed strong detachment, and only fifteen strains showed intermediate detachment. These results clearly indicate that proteins constitute a structural part of the biofilm matrix. Only one studied isolate showed >50 % detachment when biofilm was treated with sodium metaperiodate (NaIO_4_), a polysaccharide degrading agent. Many strains showed low detachment (<30 %) suggesting that polysaccharide may play a minor role in the biofilm structure of *S. haemolyticus* strains. Our study also indicated that DNAse has an effect on mature *S. haemolyticus* biofilm. Thirty-five isolates showed more than 25 % detachment of 24-h biofilm, after treatment with DNAse. Although the biofilm matrix was different in chemical composition, proteins were the main component of biofilm, in all tested strains.

**Fig. 1:**
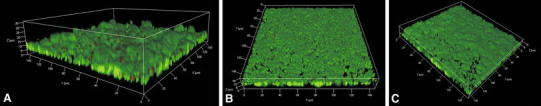
CLSM images of 24-h biofilm of *S.* *haemolyticus* MPU S 12 (**a**), MPU S 26 (**b**) and MPU S 54 (**c**) stained with SYTO9 (*green* cells) and PI (*red* cells)

The images obtained by CLSM indicate that the *S. haemolyticus* strains formed a complex 3D structure (Fig. 1). Additionally, in the mature biofilm produced by *S. haemolyticus* strains, a low number of dead cells were found. The thickness of the biofilm formed by these strains ranged from 11 to 16 μm. Apart from the differences in biofilm thickness, we also observed differences in biofilm density and the area of the growth chamber covered by these structures. However, most strains formed a biofilm in which cells were very closely packed.

The 24 strains that created the most condensed biofilm structures were chosen for further study, aiming to estimate the most effective antibiotics in treating the biofilm-associated infection caused by *S. haemolyticus* strains. When results were interpreted according to EUCAST breakpoints, planktonic forms of these strains were sensitive to rifampicin, tigecycline and daptomycin. The BIC value for tigecycline/rifampicin against cell growth in the biofilm ranged from 0.062 to 1 µg/ml, whereas the BIC for daptomycin/rifampicin was 0.125 to 2 µg/ml (Table 1). Only two isolates formed a biofilm that exhibited resistance to tigecycline (defined according to EUCAST planktonic susceptibility breakpoints). Sixteen strains of the 24 (67 %) had BIC values ≤1 µg/ml and these bacteria are categorized as susceptible to daptomycin, according to planktonic MIC (≤1 µg/ml). The results obtained in this study indicate that rifampicin in combination with tigecycline is more active against the bacteria within biofilm than daptomycin/rifampicin. The BIC value of tigecycline in combination with rifampicin was at least two to fivefold higher for 14 strains grown as a biofilm than the daptomycin/rifampicin. Nonetheless, for seven isolates, tigecycline/rifampicin was only slightly better (i.e. onefold higher) than daptomycin/rifampicin. Furthermore, daptomycin/rifampicin displayed the same effect on biofilms formed by only three isolates as tigecycline/rifampicin.

**Table 1:** In vitro antimicrobial susceptibilities of *S. haemolyticus* biofilm

BIC (µg/ml) of the following antimicrobial agents		Number of isolates
Tigecycline/rifampicin	Daptomycin/rifampicin	
0.062	2	6
0.062	1	2
0.062	0.5	2
0.125	1	2
0.125	0.5	2
0.125	0.250	3
0.125	0.125	1
0.250	0.250	2
0.5	1	2
1	2	2

## Discussion

Our study revealed that the majority of clinical *S. haemolyticus* strains have the ability to form biofilm in vitro. There is evidence that these strains form biofilm via *ica*-independent mechanisms. The results of this study are similar to previously reported data, which indicated that *icaD* genes are present only in three of the 72 tested isolates of *S. haemolyticus* [6]. According to our knowledge, the components of *S.haemolyticus* biofilm were only previously analysed by Fredheim et al. [6]. In this Norwegian study, DNAse, proteinase K and NAIO4 caused biofilm detachment for 100, 98 and 38 % of *S.haemolyticus* isolates, respectively. In our study, proteinase K caused significant detachment of bacteria in all biofilms, indicating that proteins are the predominant biofilm matrix component and play a major role in biofilm structure. This is in contrast to most other *staphylococci* species, in which PIA is the essential component of the biofilm matrix and plays a leading role in biofilm development. However, it has also been reported that *S. epidermidis* and *S. aureus* are able to form biofilm via proteinaceous process [22]. According to our study, polysaccharides, different from PIA, are also present in biofilm matrix formed by the majority of *S. haemolyticus* strains, but they play a minor role in biofilm shaping. Additionally, we also found that DNA is present in all biofilms formed by *S. haemolyticus* strains. Besides evaluation of biochemical components of the biofilm matrix, we also analysed *S. haemolyticus* biofilm structures. CLSM observation clearly demonstrated that most *S. haemolyticus* formed dense biofilm structures. These biofilms are not as thick as the PIA-dependent biofilms formed by *S. aureus* and *S. epidermidis* [24, 25].

Numerous studies indicate that biofilm-associated infections are extremely difficult to treat [13, 22, 23]. Unfortunately, only a few antimicrobials have the ability to penetrate efficiently staphylococcal biofilm and be efficient against cells in a stationary phase. It should be emphasized that the majority of studies are focused on the PIA-dependent biofilm formed by the *S. aureus* and *S. epidermidis* strains [8, 11, 17, 19, 27]. In our previous studies, we showed that the MIC value for tigecycline against *S. epidermidis* growing in the biofilm ranged from 0.125 to 2 μg/mL. Tigecycline in combination with rifampin demonstrated higher activity against bacteria embedded in biofilms than tigecycline alone [24]. To our knowledge, this is the first study to evaluate the activity of rifampicin, in combination with tigecycline, or daptomycin against proteinous biofilm formed by *S. haemolyticus.* In this study, we demonstrate that the tigecycline/rifampicin combination exhibits excellent in vitro activity against *S. haemolyticus* biofilm. All isolates, except two, formed a biofilm that exhibited susceptibility (defined according to EUCAST planktonic susceptibility breakpoints) to tigecycline. Therefore, we think that rifampicin plus tigecycline is a promising treatment option for biofilm-associated MRSH (methicilin-resistant *S. haemolyticus*) infections. Observation of an experimental rabbit model indicated that the tigecycline/rifampicin combination yielded complete clearance of MRSA infections within 3 weeks [27]. In another study by Raad et al. [19], the rifampicin/tigecycline was very effective in eliminating MRSA from a silicone disc biofilm model.

According to our studies, the combination of tigecycline/rifampicin was significantly more effective against bacteria within proteinous biofilm, than daptomycin/rifampicin. Biofilms formed by the 12 isolates of *S. haemolyticus* strains were at least three-fold more susceptible to tigecycline/rifampicin, than daptomycin/rifampicin. Nonetheless, in the case of three isolates, tigecycline/rifampicin displayed a similar effect on biofilms to daptomycin/rifampicin. Several studies have demonstrated that the effects of different combination of antimicrobial agents were strain-dependent, and for this reason, each isolate requires testing for the specific combination of the antibiotics considered for treatment [4, 23]. We think that the daptomycin/rifampicin combination can be an alternative in treating biofilm-associated infections, caused by *S. haemolyticus* strains. These combinations inhibited 67 % of biofilms, at the susceptible breakpoint of ≤1 µl/mg. Lefebvre et al. [11] showed, in a rabbit model, that daptomycin/rifampicin was able to successfully reduce bone infections caused by MRSA. Similarly, John et al. [8] demonstrated the successful treatment of guinea pig MRSA foreign-body infections with daptomycin/rifampicin. This combination therapy was significantly better than daptomycin monotherapy, in an experimental, foreign-body infections model. However, Miro et al. [14] showed that daptomycin monotherapy is more effective in the treatment of MRSA endocarditis, than daptomycin combined with rifampicin.

In conclusion, this study indicates that biofilm formation is a common phenotype among *S. haemolyticus* strains isolated from bloodstream infections. The tigecycline/rifampicin combination was more effective against *ica*-independent biofilm formed by *S. haemolyticus* strains than daptomycin/rifampicin.
